# The Effect of Bipolar Disorder Illness Course on Physiological Digital Signals: Observational Study

**DOI:** 10.2196/94737

**Published:** 2026-11-04

**Authors:** Clàudia Valenzuela-Pascual, Marina Garriga, Roger Borràs, Ruth Voltà-Solerdelcoll, Marta Korniyenko, Ariadna Mas, Clara Escala, Peter De Looff, Gerard Anmella, Antonio Benabarre, Isabella Pacchiarotti, Anna Bastidas, Isabel Agasi, Meritxell González-Campos, Iria Grande, Cristina Romero-López-Alberca, Cecilia Muñoz-Doña, Ana Catalan, Esteban Sepulveda, Eduard Vieta, Marc Valentí, Diego Hidalgo-Mazzei

**Affiliations:** 1 Department of Psychiatry and Psychology, Institute of Neuroscience, Hospital Clínic de Barcelona, Villarroel 170, Barcelona, Catalonia, 08036, Spain, 34 932275400 ext 1470; 2 Bipolar and Depressive Disorders Unit, Institut d'Investigacions Biomèdiques August Pi I Sunyer (IDIBAPS), Barcelona, Catalonia, Spain; 3 Biomedical Research Networking Centre Consortium on Mental Health (CIBERSAM), Instituto de Salud Carlos III, Madrid, Spain; 4 Department of Medicine, School of Medicine and Health Sciences, Institute of Neurosciences (UBNeuro), University of Barcelona (UB), Barcelona, Catalonia, Spain; 5 Behavioural Science Institute, Radboud University Nijmegen, Nijmegen, Gelderland, The Netherlands; 6 Fivoor, Science and Treatment Innovation, Rotterdam, The Netherlands; 7 Department of Developmental Psychology, Tilburg University, Tilburg, The Netherlands; 8 Specialized Forensic Care, De Borg National Expert Center, Den Dolder, The Netherlands; 9 Department of Psychology, University of Cádiz, Instituto de Investigación e Innovación Biomédica de Cádiz (INiBICA), Hospital Universitario Puerta del Mar, Cadiz, Andalusia, Spain; 10 Department of Neuroscience, Biobizkaia Health Research Institute, Basque Country University, Basurto University Hospital, OSI Bilbao-Basurto, Barakaldo, Bizkaia, Spain; 11 Hospital Psiquiàtric Universitari Institut Pere Mata, Reus, Spain; 12 Rovira i Virgili University, Reus, Spain; 13 Institut Investigació Sanitària Pere Virgili, CERCA, Reus, Spain

**Keywords:** bipolar disorder, digital biomarkers, electrodermal activity, heart rate, wearable electronic devices

## Abstract

**Background:**

Although bipolar disorder (BD) lacks objective biomarkers of illness progression, wearable devices enable continuous, real-world collection of physiological digital biosignals, offering a promising approach to address this gap. Measures such as electrodermal activity (EDA) and heart rate variability (HRV) fluctuate with mood state and symptom severity. Previous studies have reported reduced EDA during depressive episodes and increased HRV following symptom resolution after acute mood episodes. Furthermore, greater illness burden in BD has been associated with lower HRV, suggesting that digital biosignals may capture not only state-related changes but also aspects of illness course.

**Objective:**

This study investigated whether naturalistic EDA and heart rate (HR) profiles differ according to illness course variables in BD—including diagnosis, predominant polarity, years since onset, and lifetime number of episodes—in euthymic patients, to evaluate their potential as illness course biomarkers.

**Methods:**

This cross-sectional post hoc analysis used data from 80 euthymic patients on the BD spectrum from the INTREPIBD-TIMEBASE cohort. Participants were recruited at Hospital Clínic de Barcelona (Spain) between 2021 and 2024 and wore an Empatica E4 wristband for 48 hours in naturalistic settings. Inclusion criteria comprised a current or past *DSM-5* (*Diagnostic and Statistical Manual of Mental Disorders*, Fifth Edition) diagnosis of BD confirmed by structured interview and sustained euthymia. We examined the effects of 4 illness course variables (diagnosis, predominant polarity, years since onset, and number of lifetime episodes) on 3 biosignals (mean EDA, EDA peaks per minute, and HR) using generalized additive models. Interactions between illness course variables and circadian patterns were also assessed. All models were adjusted for age, sex, and current antipsychotic use and included a participant-specific random effect.

**Results:**

Of the 80 participants, 49 (61.3%) were female; participants’ mean age was 46.9 (SD 14.1) years. After adjustment for covariates and multiple comparisons, no significant associations were observed between illness course variables and EDA or HR. In sensitivity analyses excluding patients with schizoaffective disorder (n=75), years since illness onset and predominant polarity were associated with EDA peaks per minute after multiple comparison correction. Specifically, patients with more than 22 years since illness onset had fewer EDA peaks compared with other groups (β=−1.06, 95% CI −1.71 to −0.41; *P*=.001). No significant interactions were found between illness course variables and circadian patterns of EDA or HR.

**Conclusions:**

To our knowledge, this is the first study to examine the association between illness course variables and EDA and HR recorded in naturalistic settings in BD. In contrast to prior findings linking illness burden with HRV, our results suggest that years since illness onset may show modest associations with phasic EDA activity. These findings indicate that digital biosignals may be more sensitive to state-related changes than to illness course characteristics, limiting their use as longitudinal biomarkers of disease progression.

## Introduction

Bipolar disorder (BD) affects approximately 2.4% of individuals over their lifetime and ranks among the leading causes of disability worldwide [1]. Diagnosis and monitoring predominantly depend on clinical assessments and self-reported symptoms, resulting in a biologically and symptomatically heterogeneous population. Thus, the field requires biomarkers that offer an objective approach to tracking mental states and treatment response, potentially advancing toward personalized psychiatry [2-4]. Wearable technologies enable continuous and real-world monitoring of digital biosignals such as electrodermal activity (EDA) and heart rate (HR), which have been proposed as potential digital biomarkers [5,6].

EDA and HR are widely used psychophysiological measures. EDA primarily reflects sympathetic nervous system (SNS) activity, whereas HR reflects the balance between the SNS and the parasympathetic nervous system (PNS) [7]. EDA can be decomposed into tonic (skin conductance level [SCL]) and phasic (skin conductance response [SCR]) components, and HR can be used to derive heart rate variability (HRV) metrics. Both EDA and HR increase in response to acute stress in laboratory settings [8,9]. Evidence from naturalistic studies in BD further suggests that EDA and HR can differentiate acute mood episodes from clinical remission, as both measures change during acute episodes and then normalize with clinical remission [10,11].

However, most wearable studies in BD have focused on acute episodes or relapse prediction, with limited attention to long-term illness course variables [11,12]. The first attempt to address this long-term dimension in relation to HRV was conducted by Freyberg et al [13], who reported no HRV abnormalities in newly diagnosed BD compared with relatives and controls, suggesting that autonomic alterations may emerge with increasing illness duration. Subsequent work by Ortiz et al [14] reported that reduced HRV correlates with greater illness burden, indicating that autonomic impairment persists during euthymia and may serve as a marker of disease severity, and that increased sympathetic tone was associated with an earlier age of onset, number of lifetime depressive episodes, and a family history of suicide [15]. In a later study, the same group showed that these associations may vary by the current mood episode, with a longer duration of (hypo)manic episodes associated with increased sympathetic activation and a later age at onset of (hypo)manic episodes associated with higher parasympathetic activity [16]. In contrast, evidence on EDA in relation to illness course variables remains scarce. A systematic review reported no differences between patients with BD who were euthymic and healthy controls [17], and only one study has examined EDA reactivity in relation to long-term clinical variables, finding no association [18]. Overall, it remains unclear whether autonomic nervous system (ANS) alterations reflect enduring illness burden or whether EDA and HR measured in daily life capture meaningful markers of long-term disease course.

These uncertainties align with broader pathophysiological frameworks. BD is characterized by frequent emotional dysregulation and chronic stress [1], which may help explain the alterations in ANS activity observed in this population [19]. Another possible explanation relies on neuroprogression models that suggest that repeated mood episodes, chronic stress, and neuroinflammatory processes could lead to cumulative alterations in the ANS and other systems, which may persist even during clinical remission [20-22]. Whether wearable-derived biosignals can detect these longer-term alterations in naturalistic settings remains largely unexplored.

Considering the aforementioned findings, we hypothesized that euthymic patients with BD who exhibit indicators of a more severe illness course (in terms of illness duration, number of lifetime episodes, and diagnosis) show lower EDA in both its tonic and phasic components and higher HR compared with those with a more favorable course. Therefore, the objective of this study was to investigate whether naturalistic EDA and HR profiles differ according to illness course in euthymic patients with BD in order to evaluate their potential as illness course biomarkers.

## Methods

### Inclusion and Exclusion Criteria

The sample used for this post hoc analysis was selected from the INTREPIBD-TIMEBASE cohort [23,24]. Inclusion criteria were broad to have a representative sample of the population: (1) aged 18 to 75 years; (2) patients with a current or past diagnosis of BD according to *DSM-5* criteria and confirmed with a semistructured interview (SCID-5-RV [Structured Clinical Interview for DSM-5 Disorders, Research Version]); (3) sustained Young Mania Rating Scale (YMRS) and Hamilton Depression Rating Scale (HDRS) scores of ≤7 for at least 8 weeks; and (4) willing and able to give consent. The exclusion criteria were as follows: (1) presence of severe cardiovascular or neurological medical conditions that may lead to autonomic dysfunction, ongoing cardiovascular arrhythmia, or pacemaker use; (2) current comorbid substance use disorder as per *DSM-5* criteria, excluding nicotine substance use disorder; (3) current comorbid psychiatric disorder causing significant interference with symptoms; (4) current pharmacological treatment involving beta-blockers; and (5) ongoing pregnancy.

### Participant Characteristics

The sample consisted of 80 patients; 49 (61.3%) were female participants, with a mean age of 46.9 (SD 14.1) years (Table 1). The mean number of years since illness onset was 15.3 (SD 12.1), most had been between 2 and 5 lifetime episodes (37/75, 49.3%), and 65% (52/80) had BD type I. All participants scored very low on the YMRS and HDRS, as they needed to be euthymic for inclusion in this analysis. Of the 80 participants, 51 (63.8%) were currently receiving antipsychotic treatment and had a high level of functioning (Table 1).

**Table 1.** Demographic and clinical characteristics of the sample^a^.

Characteristics	Values
Age (y), mean (SD; n=80)	46.9 (14.1)
Sex (female; n=80), n (%)	49 (61.2)
YMRS^b^ (n=80), mean (SD)	1.5 (2.2)
HDRS^c^ (n=80), mean (SD)	3.1 (1.9)
Years since BD^d^ onset (n=80), mean (SD)	15.3 (12.1)
Number of episodes (n=75)	
First episode, n (%)	3 (4)
2 to 5	37 (49.3)
6 to 9	24 (32)
10 or more	11 (14.7)
Polarity (n=37)	
Depressive	7 (18.9)
Manic	19 (51.4)
Undetermined	11 (29.7)
Current diagnosis (n=80), n (%)	
BD I	52 (65)
BD II	17 (21.3)
BD NOS^e^	8 (10)
Schizoaffective	3 (3.8)
Antipsychotics (yes; n=80), n (%)	51 (63.8)
SOFAS^f^ (n=59), mean (SD)	76.5 (11.7)

a The table reports mean (SD) for continuous variables and n (%) for categorical variables. n in each row reports the total number of participants with available data for each variable.

b YMRS: Young Mania Rating Scale.

c HDRS: Hamilton Depression Rating Scale.

d BD: bipolar disorder.

e NOS: not otherwise specified.

f SOFAS: Social and Occupational Functioning Assessment Scale.

### Sampling Procedures, Sample Size, Power, and Precision

The sample was recruited at the Bipolar and Depressive Disorder Unit of Hospital Clínic de Barcelona between 2021 and 2024 [24]. Clinicians involved in the study invited eligible patients to participate during routine clinical visits. Thus, a nonprobability convenience sampling approach was used, based on clinician referral and patient willingness to participate. The number of individuals who declined participation was not systematically recorded. Participation was voluntary, and no financial compensation was provided. All participants were aware of the nature of the data collection, and no blinding was implemented. No a priori sample size calculation was performed, as this was a post hoc exploratory analysis of an existing cohort. From the total cohort of 362 participants, we selected 80 for this analysis. The remaining 282 were excluded due to the presence of acute symptoms (YMRS or HDRS scores >7) or did not meet the diagnostic criteria for BD.

### Ethical Considerations

This study was reviewed and approved by the Hospital Clinic of Barcelona Ethics and Research Board (HCB/2021/104). The study was also conducted in accordance with the ethical standards established in the Declaration of Helsinki and Good Clinical Practice. All participants provided written informed consent prior to inclusion in the study. All data were stored anonymously and encrypted on servers complying with the General Data Protection Regulation and the Health Insurance Portability and Accountability Act regulations. No monetary compensation was given for their participation in the study. No identifiable individual patient information is presented.

### Data Collection, Measures, Covariates, and Quality of Measurements

During an initial interview, the diagnosis was confirmed through the Structured Clinical Interview (SCID) for *DSM-5*, administered by trained mental health professionals [25]. The following clinical data were obtained from the interview and the patients’ medical records: age, sex assigned at birth, medical comorbidities, pharmacological treatment, years since BD onset, number of lifetime episodes, predominant polarity, diagnosis (BD type I, BD type II, unspecified BD, or schizoaffective disorder), and functionality (Social and Occupational Functioning Assessment Scale [SOFAS]). Pharmacological treatment was binarily categorized to reflect the presence or absence of each major pharmacological drug group, for example, current antipsychotic use. All antipsychotic medications used in the sample are reported in Table S1 in Multimedia Appendix 1. The number of lifetime episodes was grouped into clinically interpretable categories (first episode, 2‐5, 6‐9, and ≥10 episodes) to reduce recall bias and enhance data reliability during acquisition. Although initially reported by participants, episode counts were supplemented with information extracted from the electronic health record. Predominant polarity was defined as manic predominant polarity (MPP) if two-thirds or more of lifetime episodes were manic, depressive predominant polarity (DPP) if two-thirds or more of lifetime episodes were depressive, and undetermined predominant polarity (UPP) if it did not meet either of these criteria [26]. Euthymic state was defined as a score of 7 or fewer points in the Young Mania Rating Scale (YMRS) [27] and the Hamilton Depression Rating Scale (HDRS), which have well-established psychometric properties [28,29].

### Wearable Device, Preprocessing, and Data Diagnostics

Participants wore an Empatica E4 wristband (Empatica Inc) for 48 hours. The E4 is a research-grade device specifically designed and validated for ambulatory assessment of the ANS in daily life. Participants followed their daily activities and removed the wristband only when there was a risk of water contact (eg, showering and doing dishes) to avoid harming the hardware. The E4 continuously records EDA at a 4 Hz sampling rate through dry sensors and HR through a photoplethysmography sensor with a 64 Hz sampling rate. In addition, the device records triaxial acceleration (32 Hz) and skin temperature (4 Hz), which were not used in this analysis. As mentioned previously, EDA provides an established index of SNS activity and is widely used in stress and arousal research [5,30]. Cardiac measures derived from blood volume pulse include HR and HRV (and additional parameters) as an index of parasympathetic activity or balance between both branches (Task Force of the European Society of Cardiology and the North American Society of Pacing and Electrophysiology, 1996). Both HR and EDA are prone to artifacts due to sensor displacement or movement, among other reasons. A motion-artifact removal algorithm is applied by default to the HR signal; therefore, we did not add any additional filtering after extracting the data from the E4 Manager platform. Conversely, EDA is not preprocessed by Empatica and, consequently, the signal might be subject to motion artifacts and poor wrist contact, which can affect its quality. To detect artifacts and obtain clean data, we submitted the raw EDA signal to the E4 dashboard preprocessing pipeline [30-32]. The pipeline uses several quality indicators to extract clean and artifact-free data. For the current analyses, we used HR, average EDA (SCL), and nonspecific SCR (expressed as EDA peaks per minute) [33]. We specifically computed the EDA peaks per minute variable (EDA peaks) by counting the number of peaks for each minute according to the timestamp. Participants with more than 75% of artifact-contaminated signal were excluded from the analyses. Additionally, for each model, participants with missing data for the corresponding illness course variable were not included. Wearable missing data were evaluated through visual inspection, and analyses were performed using available valid data without imputation.

Statistical outliers were not explicitly defined, as extreme values were considered to have been largely addressed during the preprocessing stage through artifact removal procedures. Data distributions were visually inspected to assess plausibility and skewness. No additional data transformations were applied.

### Analytical Strategy

This study follows an observational design. To describe the sample, we present the mean (SD) for continuous variables and the number and percentage for categorical variables. To avoid collapsing EDA and HR high-granularity data into a daily mean, or forcing them into a linear model, we applied generalized additive models (GAMs) to high-level time-series data. These types of models adapt to the data by combining splines and capturing their natural form. Two types of models were performed for each outcome (mean EDA, peaks, or HR) and each illness course variable (years since onset, type of BD, number of lifetime episodes, and predominant polarity). The years since onset variable was categorized into 4 groups based on the quartile distribution observed in our sample (1‐4, 5‐13, 14‐21, and ≥22 y). This data-driven categorization allowed us to model the effect of illness duration independently of the participant-specific effects, given that each participant had a unique value for years since BD onset. By grouping this continuous variable into quartiles, we reduced sparsity and ensured sufficient sample size within each category for stable estimation. For BD type, only BD type I and II were included in the models to avoid small-group overfitting due to the small sample of nonspecified BD and schizoaffective disorder. For the predominant polarity model, participants with schizoaffective disorder were discarded as there is no official definition of this clinical course variable in this population.

In the first type of model, the dependent variable (outcome) was determined by the illness course variable, the minute of the day, and a random effect. The minute of the day was included, as both HR [34-36] and EDA [37,38] vary systematically over the course of the day, following circadian-related patterns, and the random effect represented interindividual differences. The second model included the interaction between the clinical variable and the minute of the day in order to test whether the clinical variables disrupted the circadian patterns of HR or EDA. All models were adjusted for 3 covariates: age [39], sex [40,41], and antipsychotic medication [42], because of its reported effects on HR and EDA.

We checked for concurvity between variables, especially between age and years since BD onset, as we suspected a possible correlation between them. Results showed little to no concurvity between age and years since onset, but full concordance between the random effect and age (Table S2 in Multimedia Appendix 1). All models were fitted with the bam() function from the mgcv (v 1.9‐1) package in RStudio (v. 4.4.1). Smooth terms were fitted using penalized regression splines, with smoothing parameters selected via fREML. Effective degrees of freedom were estimated during model fitting and penalized to prevent overfitting. Temporal autocorrelation was addressed using an AR(1) structure (ρ=0.9), and between-participant variability was modeled using random effects. The predicted effects of the models were illustrated with the ggplot2 (v 4.0.0) package. All analyses were conducted considering a 2-tailed type I error rate of 5% and 95% CIs. When predictors were statistically significant in the models, we performed pairwise comparisons, adjusting for multiple comparisons using the Tukey method with the emmeans package (v 1.10.5).

Lastly, we performed a sensitivity analysis excluding participants with schizoaffective disorder, as it is usually considered a different clinical entity from BD [25]. The effects of years since BD onset, number of lifetime episodes, and diagnosis were tested with GAMs against mean EDA, peaks, and HR in this sample subset.

## Results

### Clinical Predictors of EDA and HR

#### Impact of Clinical Variables on Physiological Digital Markers

Neither years since illness onset, the number of lifetime episodes, predominant polarity, nor diagnosis was associated with mean EDA, EDA peaks, or HR. Nevertheless, a longer illness duration, concretely 22 years or more since illness onset, was associated with a lower number of EDA peaks compared with patients with less than 22 years since illness onset (β=−0.81, 95% CI −1.48 to −0.34; *P*=.02; Figure 1 and Table 2), although this difference did not remain significant after multiple comparison corrections (Table S3 in Multimedia Appendix 1).

**Figure 1.**
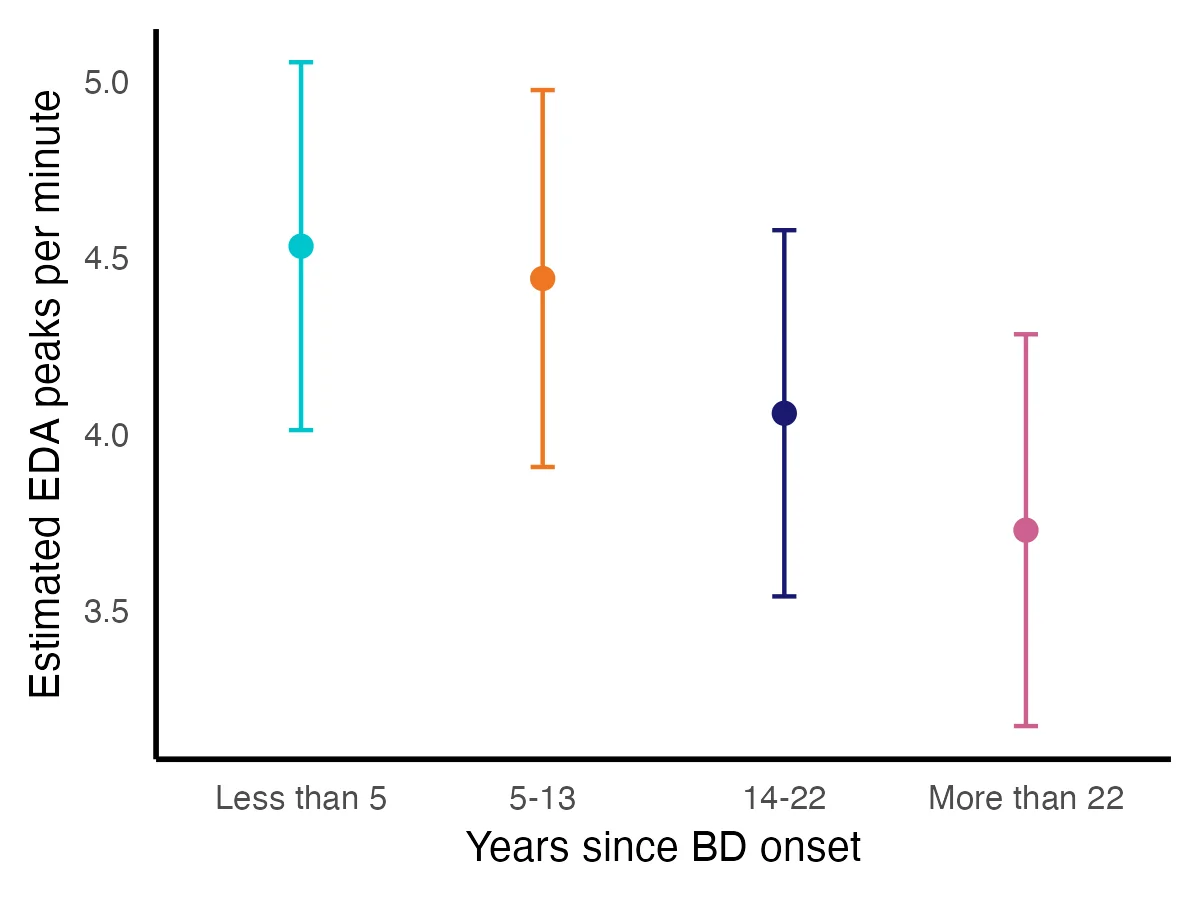
Effects of years since bipolar disorder (BD) onset on electrodermal activity (EDA) peaks per minute. The dot plot with 95% CIs shows adjusted mean estimated EDA peaks per minute across years since BD onset groups derived from a generalized additive model. The model included euthymic patients with BD and schizoaffective disorder. The analyses were conducted as part of an observational study performed at the Bipolar and Depressive Disorder Unit, Hospital Clínic de Barcelona, Spain, between 2021 and 2024. Estimates are adjusted for age, sex, and antipsychotic treatment. Error bars represent 95% CIs. No pairwise comparison was statistically significant after multiple comparison correction. The group sample sizes in each category correspond to the number of participants with valid physiological data: <5 y: 20; 5‐13 y: 20; 14‐22 y: 19; >22 y: 19.

**Table 2.** Results of the adjusted generalized additive models describing the effects of clinical variables on EDA^a^ and HR^b^ in patients with euthymic bipolar spectrum^c^.

Model and covariates	Mean EDA	*P* value	EDA peaks	*P* value	HR	*P* value
Model A, est (95% CI)						
Intercept	3.70 (2.19 to 5.21)	<.001	5.19 (4.64 to 5.75)	<.001	72.76 (66.23 to 79.30)	<.001
Years since BD^d^ onset: 5‐13 y	0.66 (−1.05 to 2.37)	.45	−0.09 (−0.71 to 0.53)	.77	−1.01 (−8.41 to 6.39)	.79
Years since BD onset: 14‐21 y	0.01 (−1.66 to 1.67)	.99	−0.47 (−1.09 to 0.15)	.13	2.18 (−5.02 to 9.39)	.55
Years since BD onset: 22 or more	−0.45 (−2.24 to 1.35)	.62	−0.81 (−1.48 to −0.13)	.02	5.54 (−2.23 to 13.30)	.16
Sex (female)	−1.26 (−2.46 to −0.06)	.04	−0.30 (−0.75 to 0.15)	.19	4.16 (−1.05 to 9.36)	.12
Antipsychotic treatment (yes)	−1.23 (−2.45 to −0.00)	.049	−0.38 (−0.84 to 0.08)	.11	5.97 (0.66 to 11.28)	.03
Smooth term, *F*(*edf*^e^)						
s(minute of the day)	9.146e+04 (9)	<.001	5.074 (6.093)	<.001	2.201e+05 (9)	<.001
s(random factor)	2.620e+09 (72)	<.001	1.524 (33.240)	.04	1.912e+07 (71.999)	<.001
s(age)	0.196 (1)	.66	0.541 (1.001)	.46	0.027 (1)	.87
n	79		78		79	
Model B, est (95% CI)						
Intercept	2.44 (−0.61 to 5.49)	.12	4.86 (3.76 to 5.96)	<.001	77.73 (63.65 to 91.82)	<.001
Number of lifetime episodes: 2 to 5	2.09 (−0.88 to 5.07)	.17	0.12 (−0.95 to 1.19)	.82	−3.36 (−17.10 to 10.38)	.63
Number of lifetime episodes: from 6 to 10	0.88 (−2.15 to 3.92)	.57	−0.25 (−1.35 to 0.84)	.65	−3.19 (−17.21 to 10.83)	.66
Number of lifetime episodes: more than 10	0.30 (−2.96 to 3.57)	.86	−0.20 (−1.42 to 1.02)	.75	−2.72 (−17.76 to 12.32)	.72
Sex (female)	−1.00 (−2.22 to 0.22)	.11	−0.18 (−0.62 to 0.27)	.44	4.18 (−1.43 to 9.78)	.15
Antipsychotic treatment (yes)	−1.37 (−2.60 to −0.13)	.03	−0.45 (−0.91 to 0.01)	.06	6.08 (0.41 to 11.75)	.04
Smooth term, *F*(*edf*)						
s(minute of the day)	9.944e+04 (9)	<.001	5.425 (6.139)	<.001	1.922e+05 (8.999)	<.001
s(random factor)	1.279e+09 (67.01)	<.001	0.805 (28.414)	<.001	1.656e+07 (66.998)	<.001
s(age)	1.367 (1)	.24	0.089 (1.001)	.77	0.293 (1)	.59
n	74		73		74	
Model C, est (95% CI)						
Intercept	4.01 (2.60 to 5.41)	<.001	5.02 (4.52 to 5.51)	<.001	73.67 (67.76 to 79.58)	<.001
BD II	0.09 (−1.43 to 1.61)	.91	−0.38 (−0.92 to 0.16)	.17	2.99 (−3.42 to 9.41)	.36
Sex (female)	−1.17 (−2.51 to 0.16)	.09	−0.23 (−0.70 to 0.24)	.33	5.87 (0.25 to 11.49)	.04
Antipsychotic treatment (yes)	−1.51 (−2.91 to −0.10)	.04	−0.47 (−0.97 to 0.03)	.06	4.93 (−1.99 to 11.84)	.10
Smooth term *F*(*edf*)						
s(minute of the day)	9.856e+04 (9)	<.001	4.708 (6.168)	<.001	2.059e+05 (8.999)	<.001
s(random factor)	2.405e+08 (63)	<.001	1.525 (27.812)	<.001	1.873e+07 (62.999)	<.001
s(age)	0.412 (1)	.52	0.001 (1.001)	.98	0.001 (1)	.97
Model D, est (95% CI)						
Intercept	4.96 (2.06 to 7.86)	<.001	5.77 (4.91 to 6.62)	<.001	70.38 (59.03 to 81.73)	<.001
MPP^f^	−0.34 (−3.13 to 2.45)	.81	−0.86 (−1.69 to −0.03)	.04	5.37 (−5.57 to 16.31)	.34
UPP^g^	−1.70 (−4.85 to 1.44)	.29	−1.62 (−2.49 to −0.75)	<.001	2.14 (−10.18 to 14.46)	.73
Sex (female)	−1.62 (−4.02 to 0.77)	.19	−0.51 (−1.20 to 0.18)	.15	3.72 (−5.67 to 13.11)	.44
Antipsychotic treatment (yes)	−1.56 (−4.02 to 0.89)	.21	−0.21 (−0.91 to 0.49)	.56	8.19 (−1.42 to 17.80)	.10
Smooth term, *F*(*edf*)						
s(minute of the day)	3.476e+04 (8.999)	<.001	0.910 (1.476)	.33	8.849e+04 (8.999)	<.001
s(random factor)	3.780e+10 (29.998)	<.001	2.732 (10.914)	.38	6.032e+07 (29.999)	<.001
s(age)	0.000 (1)	>.99	0.000 (1.000)	>.99	0.000 (1)	>.99
n	36		35		36	

a EDA: electrodermal activity.

b HR: heart rate.

c Analyses were conducted as part of an observational study conducted at the Bipolar and Depressive Disorder Unit, Hospital Clínic de Barcelona (Spain) between 2021 and 2024. Each model includes a clinical variable as the main predictor and sex, antipsychotic treatment, age, time, and a random effect as covariates. The random effect is determined by the participant’s record ID. The number of participants used for each model is indicated in the last row of each table. Estimates correspond to model coefficients. Values are presented as estimates with 95% CIs.

d BD: bipolar disorder.

e *edf*: effective degrees of freedom.

f MPP: manic predominant polarity.

g UPP: undetermined predominant polarity.

Regarding covariates, sex was only a significant predictor for HR in the diagnosis model and for mean EDA in the years-since-illness-onset model. Antipsychotic medication was a significant predictor for mean EDA in almost all models and for HR in the years-since-illness onset and lifetime-episodes models. Age was not a significant predictor in any model.

#### Interaction Between Clinical Variables and Diurnal Time

To understand whether the effect of clinical variables could intervene in EDA and HR circadian-related patterns, we examined the interactions between time and clinical variables. Models showed that the interaction with time of day was not significant for any of our illness course variables (years since BD onset, the number of lifetime episodes, predominant polarity, or type of BD) in any of the analyzed digital biosignals. Thus, this adds evidence to the fact that illness course variables do not influence HR or EDA, either independently or in a time-dependent manner (Table S4 in Multimedia Appendix 1).

### Sensitivity Analysis

When the analysis was repeated after excluding patients with schizoaffective disorder, the effect of years since illness onset on EDA peaks gained strength and precision through narrow CIs (β=−1.06, 95% CI −1.71 to −0.41; *P*=.001; Table S5A in Multimedia Appendix 1 and Figure 2). Pair-to-pair comparison revealed that the group with more than 22 years of illness had significantly fewer EDA peaks per minute than the 2 younger illness groups, even after multiple comparison correction (Table S6 in Multimedia Appendix 1). Additionally, predominant polarity significantly predicted EDA peaks, with the UPP group having significantly fewer EDA peaks than the DPP group, even after adjusting for multiple comparisons (Table S7 in Multimedia Appendix 1 and Figure 3).

**Figure 2.**
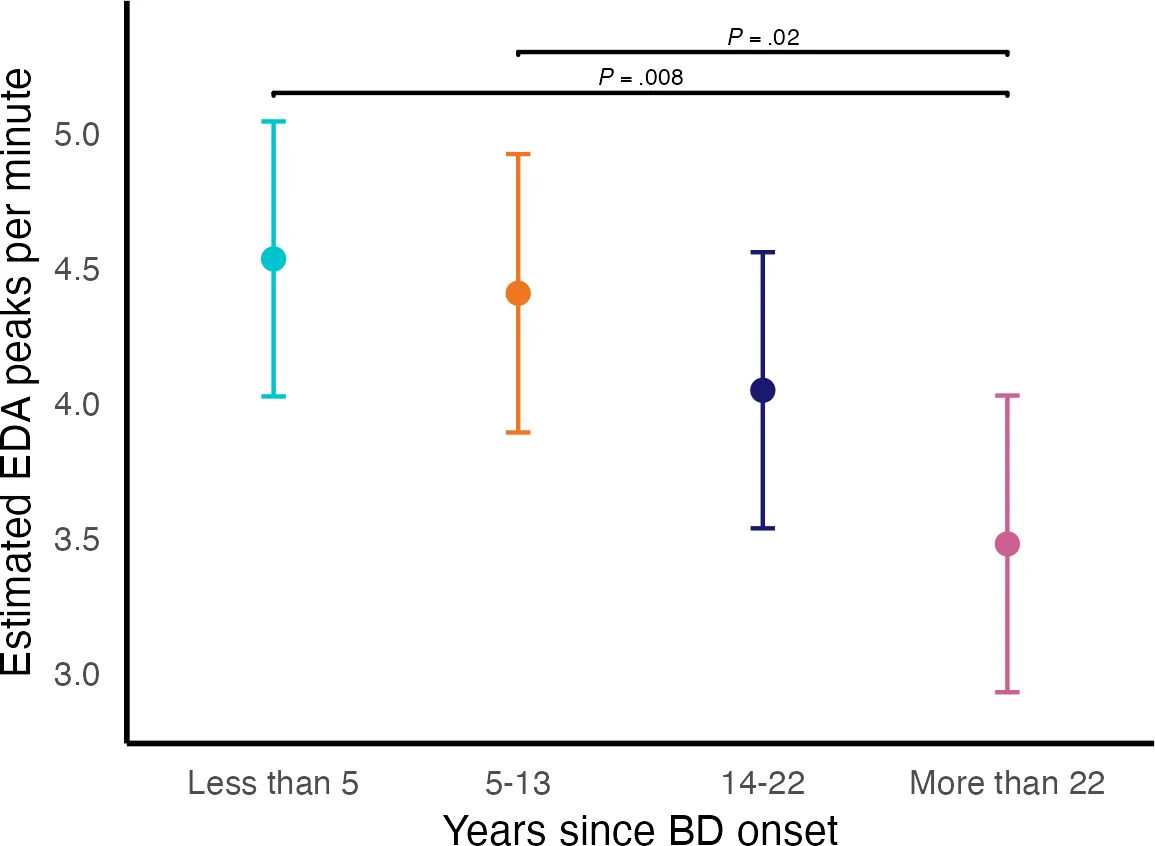
Sensitivity analysis of the association between years since bipolar disorder (BD) onset and estimated electrodermal activity (EDA) peaks per minute in the BD sample. The dot plot with 95% CIs shows adjusted mean EDA peaks per minute across years since BD onset groups derived from a generalized additive model. This model was performed for a sensitivity analysis including only euthymic patients with BD. The analyses were conducted as part of an observational study performed at the Bipolar and Depressive Disorder Unit, Hospital Clínic de Barcelona, Spain, between 2021 and 2024. Estimates were adjusted for age, sex, and antipsychotic treatment. Error bars represent 95% CIs. *P* values correspond to pairwise comparisons between groups after multiple comparison correction. Group sample sizes included in each category correspond to the number of participants with valid physiological data: less than 5 y: 19; 5‐13 y: 20; 14‐22 y: 18; more than 22 y: 18.

**Figure 3.**
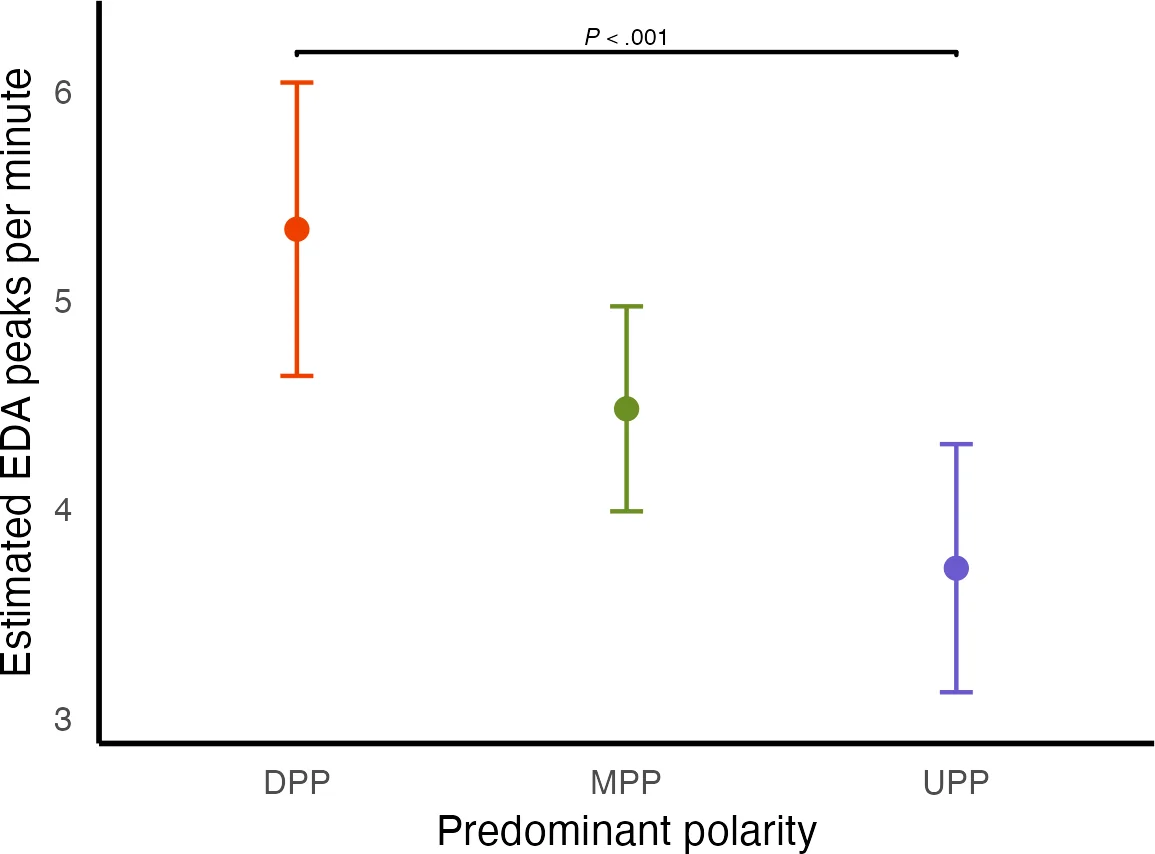
Sensitivity analysis of the association between years since bipolar disorder (BD) onset and electrodermal activity (EDA) peaks per minute in the BD-only sample. The dot plot with 95% CIs shows adjusted mean estimated EDA peaks per minute across predominant polarity groups derived from a generalized additive model. The model was performed for a sensitivity analysis including only euthymic patients with BD. The analyses were conducted as part of an observational study performed at the Bipolar and Depressive Disorder Unit, Hospital Clínic de Barcelona, Spain, between 2021 and 2024. Estimates are adjusted for age, sex, and antipsychotic treatment. Error bars represent 95% CIs. *P* values correspond to pairwise comparisons between groups after multiple comparison correction. Group sample sizes included in each category correspond to the number of participants with valid physiological data: depressive predominant polarity (DPP): 7; manic predominant polarity (MPP): 18; undetermined predominant polarity (UPP): 10.

## Discussion

### Principal Findings

This exploratory, post hoc, cross-sectional study investigated whether BD illness course variables (years since illness onset, diagnosis, number of lifetime episodes, and predominant polarity) predicted EDA and HR in euthymic patients. Using GAMs, the main results were negative; however, a trend emerged suggesting that longer illness duration was linked to fewer EDA peaks per minute. This trend became statistically significant when excluding patients with schizoaffective disorder, pointing to a potential cumulative dampening of nonspecific SCR over time. Lastly, the absence of significant effects of the 4 analyzed clinical variables on HR and the circadian-related patterns of EDA and HR further supports the limited influence of these illness course variables on digital biosignals. Altogether, our findings suggest that the influence of BD illness course on EDA and HR in naturalistic settings is negligible, supporting the view that these measures could function primarily as state-dependent biomarkers that capture transient physiological fluctuations rather than stable illness traits.

To our knowledge, no previous studies have examined EDA in euthymic patients with BD under naturalistic conditions. In contrast, there is prior evidence showing associations between illness duration and HRV [14,43]. Although HR and HRV are closely related, their relationship is nonlinear; it has been described that in conditions of repeated exposure to stress, HR rises and HRV decreases [44,45]. Prior orthostatic testing observed that older age at onset correlated with lower very-low-frequency and high-frequency bands, reflecting reduced ANS activation [46]. However, this correlation arose only during the third position of an orthostatic test, which corresponds to the moment when the patient lies down after standing for 5 minutes. Thus, it arose under artificial testing conditions rather than in naturalistic environments [46]. In naturalistic settings, Ortiz et al [14] also reported a negative association between HRV, specifically root mean square of successive differences and the SD of all the normal R-R intervals, and age at BD onset [14]. Importantly, while previous work has focused exclusively on HRV, our study is the first to examine whether HR itself varies with illness course indicators in everyday conditions, providing novel evidence that HR does not show these associations in euthymic patients with BD.

Our negative findings regarding the associations between the analyzed biosignals and diagnosis and number of episodes cannot be compared with previous studies because, as far as we know, no other work has tested these variables. However, the ANS typically responds to stress and arousal, which are states, and is not as sensitive to traits, not even to sex in some studies [12,47]. Consequently, it is plausible that diagnosis, which reflects clinical phenotypes that influence behavior and symptomatology, does not substantially affect ANS functioning per se. In addition, the number of lifetime episodes could be considered a reasonable proxy for neuroprogression, as this theory is based on the presumed toxicity of episodes [22]. However, it does not capture episode intensity or duration and relies primarily on patient recall; therefore, it is not very robust, and in addition, extreme groups are underrepresented in our sample. By contrast, years since BD onset is more objective and captures the physiological load accumulated over time. Overall, our predominantly negative findings support the notion that digital biosignals are state-dependent rather than trait-dependent, following the trend observed in other peripheral biomarkers, such as inflammatory markers and neurotrophic factors, which tend to normalize during euthymic phases in BD, reaching levels similar to those of healthy controls [48-51]. From a clinical perspective, this reinforces the potential role of wearable-derived digital biosignals as tools for monitoring dynamic changes in mental states and relapse risk, rather than cross-sectional indicators of illness severity [11,12,52,53].

We also did not find any interaction between illness course variables and the biosignals’ circadian patterns.. HR and tonic EDA circadian patterns are highly stable due to their dependence on core biological factors such as the sleep-wake cycle, hormonal rhythms, and thermoregulation [54]. As these remain stable, the influence of a long-term clinical factor could become negligible. This would explain why, in our models, the effect of minute of day on EDA and HR is large, while the effects of clinical variables, if present at all, were small (Tables S4-S7 in Multimedia Appendix 1). Moreover, previous studies have only reported alterations in the mean or basal tone of HR or EDA, but not in their circadian patterns [10,14]. Thus, circadian patterns of sympathetic arousal, indexed by EDA and cardiac activity, remain largely preserved and primarily driven by sleep-wake cycles and central circadian mechanisms, which likely explains the absence of significant clinical and time-of-day interactions [55].

From the sensitivity analyses, we observed that excluding participants with schizoaffective disorder strengthened the group differences. Specifically, the group with more than 22 years of BD onset showed significantly fewer EDA peaks per minute than the group with less than 13 years since onset. The consistency of findings across sensitivity analyses suggests a modest effect of clinical heterogeneity on EDA. In addition, the predominant polarity model reached statistical significance, with the UPP group displaying fewer EDA peaks than the DPP group. However, we do not believe these findings reflect cross-sectional diagnostic differences but rather the clinical characteristics of these specific participants. Moreover, the predominant polarity model had the lowest statistical power, as this variable was available for only 35 participants. Thus, although the UPP group exhibited fewer EDA peaks than the DPP group, even after adjusting for multiple comparisons, these results warrant replication in a larger sample. Predominant polarity was obtained primarily relying on patient recall, making it particularly vulnerable to recall and cognitive biases and, therefore, the least reliable of the 4 illness course variables explored. Lastly, the predominant polarity groups did not differ significantly in sex, years since BD onset, or antipsychotic use (Table S8 in Multimedia Appendix 1).

The association we observed between longer illness duration and fewer EDA peaks per minute could be explained by several underlying mechanisms. First, habituation refers to the gradual reduction in physiological responsiveness that occurs when repeated or prolonged sympathetic activation leads the system to produce weaker reactions over time [43]. If we understand BD as a possible cause of repeated and prolonged sympathetic activation [44], habituation could explain the reduction in skin conductance reactivity and, therefore, the fewer EDA peaks reported in our sample. This mechanism is known as allostatic load, whereby prolonged exposure to stress leads to a lower phasic response and a higher basal tone [45,46]. Second, long-term antipsychotic use or chronic benzodiazepine intake can attenuate sympathetic activity [47,48]. Although we controlled for current antipsychotic use, we were unable to control for cumulative antipsychotic exposure across the lifetime. Third, the findings could also be attributed to neuroprogression hypotheses within BD staging frameworks [49]. Focusing on Berk’s staging proposal, which emphasizes episode number, as opposed to Kapczinski’s model, which prioritizes functional impairment, the repetition of episodes may induce biological changes in frontolimbic networks, oxidative stress, and inflammation [22,50]. However, this neuroprogression effect should be more clearly detected through the number of lifetime episodes rather than years since onset, as it is postulated that the number of episodes is what drives these changes, but this effect was not observed in our data.

### Strengths and limitations

Our study has several limitations. First, although restricting the sample to euthymic patients is justified, it also introduces methodological constraints. Selecting only euthymic patients reduces the variability of HR and EDA signals, as these mainly fluctuate during acute states. This reduced variability may limit the ability to detect associations with illness course variables, particularly if their effects are small. Additionally, if the impact of illness course variables is primarily expressed during acute states, as previous literature suggests [16], analyzing only euthymic patients may further reduce the capacity to detect such effects. Finally, our design precludes examining potential interactions between illness course variables and mood states.

Another limitation was the categorization of years since illness onset into quartiles, which may result in loss of information and reduced variability. However, this approach allowed more stable group comparisons and improved clinical interpretability, and we were still able to detect an effect of this variable. Additionally, the post hoc exploratory design may increase the probability of false-positive findings because the hypothesis is not predefined [56]. Although models were adjusted for current antipsychotic treatment, other potential confounding factors, such as lifetime pharmacological exposure, cumulative medication effects, and comorbid medical conditions, were not systematically accounted for. These factors may independently influence autonomic nervous system activity and could therefore partially explain the observed associations. The relatively small sample size in some subgroup analyses and the short recording period (48 h) may further limit the robustness of the findings. In particular, short monitoring durations may not fully capture intraindividual variability in physiological signals, especially in BD, where mood episodes evolve over longer timescales, such as 2 weeks for depressive episodes. Consequently, these factors may reduce the ability to detect subtle physiological patterns and limit the generalizability of the results. Our analysis focused on the tonic and phasic components of EDA and HR, but including HRV metrics and other physiological or behavioral digital biosignals may provide complementary information [57]. Finally, the lack of external validation of our models and the absence of comparable methodologies in the literature hinder direct comparison with other studies.

On the other hand, our study has several strengths, such as the use of high-resolution, real-world physiological data collected via wearable devices. Wearables allow a granular and objective assessment of physiological digital biosignals that is easy to implement in real clinical settings [52,58]. Also, the application of advanced statistical methods, such as GAMs, enabled us to capture nonlinear relationships and circadian-related patterns in the data. Additionally, the inclusion of a diverse set of clinical variables (number of episodes, diagnosis, predominant polarity, and years since onset) allowed for a comprehensive exploration of illness course and its impact on digital biosignals.

### Future directions

On the other hand, our study has several strengths, such as the use of high-resolution, real-world physiological data collected via wearable devices. Wearables allow a granular and objective assessment of physiological digital biosignals that is easy to implement in real clinical settings [52,58]. Also, the application of advanced statistical methods, such as GAMs, enabled us to capture nonlinear relationships and circadian-related patterns in the data. Additionally, the inclusion of a diverse set of clinical variables (number of episodes, diagnosis, predominant polarity, and years since onset) allowed for a comprehensive exploration of illness course and its impact on digital biosignals.

Future research should aim to replicate and extend our findings to determine whether BD illness course variables truly have no meaningful impact on ANS functioning, as our data suggest. The relationship between HRV and illness course variables should also be further explored. Additionally, although illness duration was associated with physiological signatures compatible with greater allostatic load, this finding neither confirms nor refutes the neuroprogression hypothesis, and rigorously designed longitudinal studies explicitly targeting neuroprogressive mechanisms are still needed to address this question. In parallel, efforts should shift toward more precise characterization of the specific states to which digital biosignals such as EDA and HR are most sensitive, so they can be effectively integrated into clinical management of BD and other conditions where autonomic arousal is relevant. A state-focused approach may ultimately enable the development of transdiagnostic, stress-tailored interventions that improve functioning and long-term outcomes.

### Conclusions

Our findings support a predominantly state-dependent role of digital biosignals in BD and suggest that, in clinically stable patients, EDA and HR alone may have limited value as cross-sectional indicators of illness course, although subtle autonomic alterations associated with years since illness onset cannot be excluded and warrant confirmation in larger, longitudinal studies [2,3,52,59]. Despite the limitations, our study demonstrates the potential of wearable technologies for objective, real-world monitoring in BD, specifically as state markers. Finally, we emphasize the importance of further research to rigorously validate these digital biosignals and support their translation into clinically relevant biomarkers [52].

## Supplementary material

10.2196/94737 Multimedia Appendix 1 Supplementary details on antipsychotic drugs, concurvity, pairwise comparisons, electrodermal activity, and heart rate models including time interaction, sensitivity analyses, and additional descriptive group comparisons by predominant polarity.

## Abbreviations

ANS: autonomic nervous system
BD: bipolar disorder
DPP: depressive predominant polarity
*DSM-5*: *Diagnostic and Statistical Manual of Mental Disorders*, Fifth Edition
EDA: electrodermal activity
GAM: generalized additive model
HDRS: Hamilton Depression Rating Scale
HR: heart rate
HRV: heart rate variability
MPP: manic predominant polarity
PNS: parasympathetic nervous system
SCID: Structured Clinical Interview
SCID-5-RV: Structured Clinical Interview for DSM-5 Disorders, Research Version
SCL: skin conductance level
SCR: skin conductance response
SNS: sympathetic nervous system
SOFAS: Social and Occupational Functioning Assessment Scale
UPP: undetermined predominant polarity
YMRS: Young Mania Rating Scale
