# Supplementary material for: The Effect of Bipolar Disorder Illness Course on Physiological Digital Signals: Observational Study
Source: JMIR Ment Health. 2026 Nov 4;13:e94737. doi: 10.2196/94737 (PMC13734704; doi:10.2196/94737)
Supplement: Supplementary file 1 [file mental-v13-e94737-s001.docx]

# Multimedia Appendix 1

**Index**

[SUPPLEMENTARY MATERIAL	1](#_Toc806000125)

[Supplementary Table 1: Generic name of antipsychotic drugs.	1](#_Toc134628798)

[Supplementary Table 2: Concurvity analysis:	2](#_Toc459128722)

[Supplementary Table 3: Pairwise comparisons of EDA peaks across years since illness onset.	3](#_Toc1649617764)

[Supplementary Table 4: Generalized Additive Mixed Models of HR and EDA including the interaction between clinical variables and time of the day.	3](#_Toc1535265736)

[Supplementary Table 5: Sensitivity analysis Effects of clinical variables on EDA and HR only on BD sample.	5](#_Toc1895242512)

[Supplementary Table 6: Sensitivity analysis, pairwise comparisons of EDA peaks across years since BD onset.	6](#_Toc1855894997)

[Supplementary Table 7: Sensitivity analysis, pairwise comparisons of EDA peaks across polarity.	7](#_Toc259768125)

[Supplementary Table 8: Group differences according to predominant polarity.	7](#_Toc1848978326)

**Supplementary Table S1: Generic name of antipsychotic drugs.**

The table includes the number of participants that were under its treatment during the study and its percentage over the total of 80 participants.

| Antipsychotic medication | n | % |
| --- | --- | --- |
| Aripiprazole | 12 | 15% |
| Asenapine | 7 | 8.8% |
| Cariprazine | 1 | 1.3% |
| Clozapine | 3 | 3.8% |
| Lurasidone | 9 | 11.3% |
| Olanzapine | 8 | 10% |
| Paliperidone | 4 | 5% |
| Quetiapine | 20 | 25% |
| Clotiapine | 2 | 2.5% |
| Haloperidol | 1 | 1.3% |
| Perphenazine | 1 | 1.3% |

**Supplementary Table S2: Concurvity analysis.**

Results extracted for one of the models, but all of them had similar values as the presented variables remain the same in all models. Worst, observed and estimate refer to three methods of computing concurvity between smooth estimates.

|  | S(minute of day) | S(random factor) | S(age) |
| --- | --- | --- | --- |
| Worst |  |  |  |
| S(minute of day) | 1.00 | 0.09 | 0.02 |
| S(random factor) | 0.09 | 1.00 | 1.00 |
| S(age) | 0.02 | 1.00 | 1.00 |
| Observed |  |  |  |
| S(minute of day) | 1.00 | 0.00 | 0.01 |
| S(random factor) | 0.08 | 1.00 | 1.00 |
| S(age) | 0.01 | 0.11 | 1.00 |
| Estimate |  |  |  |
| S(minute of day) | 1.00 | 0.00 | 0.01 |
| S(random factor) | 0.07 | 1.00 | 1.00 |
| S(age) | 0.01 | 0.15 | 1.00 |

**Supplementary Table S3: Pairwise comparisons of EDA peaks across years since illness onset.**

Pairwise differences of estimated marginal means (EMMs) for years since illness onset. Positive estimates indicate higher mean in the first‑listed group. EMMs were obtained with emmeans and Tukey adjustment for a family of 4 estimates; results are averaged over the levels of sex and antipsychotic treatment. Statistical significance was set at α = .05; bold rows denote P < 0.05.

| **Contrast** | **Estimate (± SE)** |  | **It ratio** | **P value (Tukey)** |
| --- | --- | --- | --- | --- |
| Less than 5 vs 5–13 | 0.09 ± 0.31 | 118,161 | 0.288 | 0.99 |
| Less than 5 vs 14–21 | 0.47 ± 0.32 | 118,161 | 1.497 | 0.44 |
| Less than 5 vs more than 22 | 0.80 ± 0.34 | 118,161 | 2.338 | 0.09 |
| 5–13 vs 14–21 | 0.38 ± 0.32 | 118,161 | 1.195 | 0.63 |
| 5–13 vs more than 22 | 0.71 ± 0.33 | 118,161 | 2.179 | 0.13 |
| 14–21 vs more than 22 | 0.33 ± 0.33 | 118,161 | 1.006 | 0.75 |

**Supplementary Table S4: Generalized Additive Mixed Models of HR and EDA including the interaction between clinical variables and time of the day.**

Each model includes a clinical variable as the main predictor and sex, antipsychotic treatment, age, time and a random effect as covariates, and an interaction term between the clinical covariates and time of the day. The random effect is determined by the subject’s record id. The number of subjects used for each model is indicated in the last row of each table.

Abbreviations: BD: Bipolar disorder, EDA: electrodermal activity, HR: heart rate, est: estimate, SE: standard error, P: p-value, CI: Confidence intervals, edf: estimated degrees of freedom, MPP: manic predominant polarity, UPP: undetermined predominant polarity.

| **A** | **EDA mean (est ± SE)** | | **P** | **EDA peaks (est ± SE)** | **P** | **HR (est ± SE)** | **P** |
| --- | --- | --- | --- | --- | --- | --- | --- |
| **Intercept** | 3.782 ± 0.773 | <0.001*** | | 5.191 ± 0.284 | <0.001*** | 73.073 ± 3.313 | <0.001*** |
| **Years since BD onset: 5–13** | 0.528 ± 0.875 | 0.546 | | −0.092 ± 0.318 | 0.774 | −1.206 ± 3.737 | 0.747 |
| **Years since BD onset: 14–21** | −0.001 ± 0.852 | 0.999 | | −0.474 ± 0.317 | 0.135 | 2.175 ± 3.639 | 0.550 |
| **Years since BD onset: 22 or more** | −0.592 ± 0.919 | 0.519 | | −0.805 ± 0.344 | 0.020* | 4.510 ± 3.924 | 0.251 |
| **Sex (female)** | −1.275 ± 0.616 | 0.038* | | −0.299 ± 0.230 | 0.193 | 4.126 ± 2.628 | 0.117 |
| **Antipsychotic treatment (yes)** | −1.223 ± 0.628 | 0.051. | | −0.376 ± 0.235 | 0.110 | 5.932 ± 2.680 | 0.027* |
| **Smooth term** | **EDA mean F(edf)** | **P** | | **EDA peaks F(edf)** | **P** | **HR F(edf)** | **P** |
| **s(minute of the day)** | 8.943 (1) | 0.0028 ** | | 5.070 (6.09) | <0.001*** | 38.47 (1.002) | <0.001*** |
| **s(interaction)** | 1.24e14 (35) | 1.000 | | 0.000 (0.01) | 0.951 | 1.22e9 (34.99) | 1.000 |
| **s(random factor)** | 3.49e9 (72) | <0.001*** | | 2.572 (33.24) | 0.0098 ** | 3.73e7 (72) | <0.001*** |
| **s(age)** | 0.205 (1) | 0.651 | | 0.540 (1.002) | 0.462 | 0.030 (1) | 0.863 |
| **N** | 79 |  | | 78 |  | 79 |  |

| **B** | **EDA mean (est ± SE)** | **P** | **EDA peaks (est ± SE)** | **P** | **HR (est ± SE)** | **P** |
| --- | --- | --- | --- | --- | --- | --- |
| **Intercept** | 2.609 ± 1.555 | 0.093. | 4.913 ± 0.564 | <0.001*** | 77.644 ± 7.189 | <0.001*** |
| **Number of lifetime episodes: 2 to 5** | 1.833 ± 1.518 | 0.227 | 0.123 ± 0.548 | 0.823 | −3.227 ± 7.016 | 0.646 |
| **Number of lifetime episodes: from 6 to 10** | 0.672 ± 1.549 | 0.664 | −0.251 ± 0.558 | 0.653 | −3.167 ± 7.156 | 0.658 |
| **Number of lifetime episodes: more than 10** | 0.106 ± 1.664 | 0.950 | −0.196 ± 0.625 | 0.754 | −2.686 ± 7.682 | 0.727 |
| **Sex (female)** | −0.992 ± 0.623 | 0.111 | −0.178 ± 0.228 | 0.435 | 4.197 ± 2.864 | 0.143 |
| **Antipsychotic treatment (yes)** | −1.376 ± 0.629 | 0.029* | −0.447 ± 0.235 | 0.057. | 6.104 ± 2.893 | 0.035* |
| **Smooth term** | **EDA mean F(edf)** | **P** | **EDA peaks F(edf)** | **P** | **HR F(edf)** | **P** |
| **s(minute of the day)** | 2.377 (1.00) | 0.123 | 5.015 (6.072) | <0.001*** | 1.036 (1.00) | 0.342 |
| **s(interaction)** | 3.41e10 (34.99) | 1.000 | 0.000 (0.009) | 0.903 | 9.837e09 (34.97) | 1.000 |
| **s(random factor)** | 2.889e09 (67.01) | <0.001*** | 1.863 (28.423) | 0.041* | 3.010e07 (67.00) | <0.001*** |
| **s(age)** | 1.389 (1.00) | 0.239 | 0.087 (1.001) | 0.769 | 0.310 (1.00) | 0.577 |
| **N** | 74 |  | 73 |  | 74 |  |

| **C** | **EDA mean (est ± SE)** | **P** | **EDA peaks (est ± SE)** | **P** | **HR (est ± SE)** | **P** |
| --- | --- | --- | --- | --- | --- | --- |
| **BD I (Intercept)** | 3.903 ± 0.719 | <0.001*** | 5.023 ± 0.252 | <0.001*** | 73.606 ± 3.026 | <0.001*** |
| **BD II** | 0.312 ± 0.774 | 0.687 | −0.421 ± 0.277 | 0.129 | 3.107 ± 3.282 | 0.344 |
| **Sex** | −1.160 ± 0.679 | 0.088. | −0.236 ± 0.240 | 0.326 | 5.868 ± 2.878 | 0.042* |
| **Antipsychotic treatment (ap_2nd)** | −1.496 ± 0.714 | 0.036* | −0.465 ± 0.255 | 0.068. | 4.928 ± 3.028 | 0.104 |
| **Smooth term** | **EDA mean F(edf)** | **P** | **EDA peaks F(edf)** | **P** | **HR F(edf)** | **P** |
| **s(minute of the day)** | 0.723 (1.003) | 0.396 | 4.002 (5.959) | 0.000247 *** | 0.001 (1.012) | 0.996 |
| **s(interaction)** | 9.55e11 (16.996) | 1.000 | 0.223 (1.515) | 0.093 | 1.94e09 (16.984) | 1.000 |
| **s(random factor)** | 8.44e08 (63.001) | <0.001*** | 1.778 (27.793) | 0.003 ** | 7.13e06 (62.999) | <0.001*** |
| **s(age)** | 0.408 (1.000) | 0.523 | 0.001 (1.002) | 0.992 | 0.001 (1.000) | 0.979 |
| **N** | 68 |  | 67 |  | 68 |  |

| **D** | **EDA mean (est ± SE)** | **P** | **EDA peaks (est ± SE)** | **p** | **HR (est ± SE)** | **p** |
| --- | --- | --- | --- | --- | --- | --- |
| **DPP (Intercept)** | 4.986 ± 1.492 | <0.001*** | 5.764 ± 0.436 | <0.001*** | 70.287 ± 5.769 | <0.001*** |
| **MPP** | −0.387 ± 1.434 | 0.787 | −0.860 ± 0.425 | 0.043* | 5.598 ± 5.545 | 0.313 |
| **UPP** | −1.791 ± 1.615 | 0.267 | −1.624 ± 0.445 | <0.001*** | 1.944 ± 6.245 | 0.756 |
| **Sex** | −1.629 ± 1.231 | 0.186 | −0.511 ± 0.354 | 0.148 | 3.669 ± 4.764 | 0.441 |
| **Antipsychotic treatment (ap_2nd)** | −1.586 ± 1.259 | 0.208 | −0.211 ± 0.359 | 0.556 | 8.185 ± 4.872 | 0.093. |
| **Smooth term** | **EDA mean F(edf)** | **P** | **EDA peaks F(edf)** | **P** | **HR F(edf)** | **P** |
| **s(minute of the day)** | 5.560 (1.000) | 0.018* | 0.909 (1.477) | 0.325 | 76.58 (1.001) | <0.001*** |
| **s(interaction)** | 9.902e+06 (26.000) | <0.001*** | 0.000 (0.003) | 0.683 | 3.177e+05 (25.991) | <0.001*** |
| **s(random factor)** | 7.463e+06 (30.000) | <0.001*** | 0.599 (10.914) | 0.014* | 3.657e+04 (30.000) | <0.001*** |
| **s(age)** | 1.014 (1.000) | 0.314 | 0.041 (1.000) | 0.839 | 1.201 (1.000) | 0.273 |
| **N** | 36 |  | 35 |  | 36 |  |

**Supplementary Table S5: Sensitivity analysis effects of clinical variables on EDA and HR only on BD sample.**

Each model includes a clinical variable as the main predictor and sex, antipsychotic treatment, age, time and a random effect as covariates. The random effect is determined by the subject’s record id. The number of subjects used for each model is indicated in the last row of each table.

Abbreviations: BD: Bipolar disorder, EDA: electrodermal activity, HR: heart rate, est: estimate, SE: standard error, P: p-value, CI: Confidence intervals, edf: estimated degrees of freedom.

| **A** | **EDA mean (est ± SE)** | **P** | **EDA peaks (est ± SE)** | **P** | **HR (est ± SE)** | **P** |
| --- | --- | --- | --- | --- | --- | --- |
| **Intercept** | 3.757 ± 0.790 | <0.001*** | 5.212 ± 0.267 | <0.001*** | 72.847 ± 3.393 | <0.001*** |
| **Years since BD onset: 5–13** | 0.661 ± 0.894 | 0.460 | −0.127 ± 0.300 | 0.672 | −1.451 ± 3.834 | 0.705 |
| **Years since BD onset: 14–21** | −0.084 ± 0.879 | 0.924 | −0.486 ± 0.302 | 0.108 | 1.960 ± 3.774 | 0.603 |
| **Years since BD onset: 22 or more** | −0.630 ± 0.950 | 0.507 | −1.055 ± 0.331 | 0.0014 ** | 6.084 ± 4.077 | 0.136 |
| **Sex (female)** | −1.199 ± 0.637 | 0.060. | −0.167 ± 0.221 | 0.450 | 3.965 ± 2.733 | 0.147 |
| **Antipsychotic treatment (yes)** | −1.344 ± 0.641 | 0.036* | −0.474 ± 0.223 | 0.033* | 6.618 ± 2.752 | 0.016* |
| **Smooth term** | **EDA mean F(edf)** | **P** | **EDA peaks F(edf)** | **P** | **HR F(edf)** | **P** |
| **s(minute of the day)** | 9.409e+04 (9) | <0.001*** | 5.033 (6.282) | <0.001*** | 2.071e+05 (8.999) | <0.001*** |
| **s(random factor)** | 2.855e+09 (69) | <0.001*** | 0.931 (26.763) | 0.059. | 1.593e+07 (68.999) | <0.001*** |
| **s(age)** | 0.246 (1) | 0.620 | 0.749 (1.001) | 0.387 | 0.081 (1) | 0.776 |
| **N** | 76 |  | 75 |  | 76 |  |

| **B** | **EDA mean (est ± SE)** | **P** | **EDA peaks (est ± SE)** | **P** | **HR (est ± SE)** | **P** |
| --- | --- | --- | --- | --- | --- | --- |
| **Intercept** | 2.490 ± 1.577 | 0.114 | 4.850 ± 0.575 | <0.001*** | 77.345 ± 7.260 | <0.001*** |
| **Number of lifetime episodes: 2 to 5** | 2.101 ± 1.539 | 0.172 | 0.122 ± 0.558 | 0.827 | −3.016 ± 7.079 | 0.670 |
| **Number of lifetime episodes: from 6 to 10** | 0.803 ± 1.573 | 0.610 | −0.243 ± 0.570 | 0.670 | −3.026 ± 7.233 | 0.676 |
| **Number of lifetime episodes: more than 10** | 0.299 ± 1.685 | 0.859 | −0.195 ± 0.636 | 0.759 | −2.607 ± 7.742 | 0.736 |
| **Sex (female)** | −1.028 ± 0.637 | 0.106 | −0.179 ± 0.234 | 0.446 | 4.513 ± 2.914 | 0.122 |
| **Antipsychotic treatment (yes)** | −1.390 ± 0.639 | 0.030* | −0.447 ± 0.240 | 0.063. | 6.265 ± 2.926 | 0.032* |
| **Smooth term** | **EDA mean F(edf)** | **P** | **EDA peaks F(edf)** | **P** | **HR F(edf)** | **P** |
| **s(minute of the day)** | 9.323e+04 (9) | <0.001*** | 5.073 (6.207) | <0.001*** | 1.820e+05 (8.999) | <0.001*** |
| **s(random factor)** | 2.984e+08 (65.01) | <0.001*** | 0.851 (28.359) | <0.001*** | 6.315e+06 (64.998) | <0.001*** |
| **s(age)** | 1.372 (1) | 0.241 | 0.079 (1.000) | 0.780 | 0.173 (1) | 0.677 |
| **N** | 72 |  | 71 |  | 72 |  |

**Supplementary Table S6: Sensitivity analysis, pairwise comparisons of EDA peaks across years since BD onset.**

Pairwise differences of estimated marginal means (EMMs) for years since illness onset. Positive estimates indicate higher mean in the first‑listed group. EMMs were obtained with emmeans and Tukey adjustment for a family of 4 estimates; results are averaged over the levels of sex and antipsychotic treatment. Statistical significance was set at α = .05; bold rows denote P < 0.05.

Abbreviations: SE: standard error, df: degrees of freedom, P: p-value.

| **Contrast** | **Estimate (± SE)** | **df** | **t ratio** | **P (Tukey)** |
| --- | --- | --- | --- | --- |
| Less than 5 vs 5–13 | 0.127 ± 0.300 | 112,669 | 0.424 | 0.9744 |
| Less than 5 vs 14–21 | 0.486 ± 0.302 | 112,669 | 1.608 | 0.3737 |
| Less than 5 vs more than 22 | **1.055 ± 0.331** | **112,669** | **3.186** | **0.0079***** |
| 5–13 vs 14–21 | 0.359 ± 0.305 | 112,669 | 1.177 | 0.6411 |
| 5–13 vs more than 22 | **0.928 ± 0.312** | **112,669** | **2.971** | **0.0157*** |
| 14–21 vs more than 22 | 0.569 ± 0.319 | 112,669 | 1.785 | 0.2804 |

**Supplementary Table S7: Sensitivity analysis, pairwise comparisons of EDA peaks across polarity.**

Pairwise differences of estimated marginal means (EMMs) for polarity. Positive estimates indicate a higher mean in the first‑listed group. EMMs were obtained with emmeans, and P values are Tukey‑adjusted for a family of 3 comparisons; results are averaged over sex and antipsychotic treatment. Statistical significance was set at α = 0.05; bold rows denote P < 0.05.

Abbreviations: SE: standard error, df: degrees of freedom, P: p-value.

| **Contrast** | **Estimate (± SE)** | **df** | **t ratio** | **P (Tukey)** |
| --- | --- | --- | --- | --- |
| DPP vs MPP | 0.860 ± 0.425 | 50,794 | 2.024 | 0.1066 |
| **DPP vs UPP** | **1.624 ± 0.445** | **50,794** | **3.652** | **0.0008***** |
| MPP vs UPP | 0.763 ± 0.370 | 50,794 | 2.065 | 0.0973 |

**Supplementary Table S8: Group differences according to predominant polarity.**

Cell values represent the number of individuals in each category. Chi-square tests were performed using Monte Carlo simulated p-values (1,000,000 replicates) due to low expected cell frequencies. No correction for multiple comparisons was applied, as the analyses were exploratory, and p values were not significant in the first round. Abbreviations: DPP, depressive predominant polarity; MPP, manic predominant polarity; UPP, undetermined predominant polarity, P: p-value, **χ²**: Chi-square statistic.

|  |  | **Predominant polarity** | | |  |  |  |
| --- | --- | --- | --- | --- | --- | --- | --- |
|  |  | **DPP** | **MPP** | **UPP** | **Sum** | **χ²** | **‑P** |
| **Sex** | Female | 4 | 8 | 5 | 17 |  |  |
|  | Male | 3 | 11 | 6 | 20 | 0.47 | 0.91 |
| **Antipsychotic use** | Yes | 5 | 12 | 8 | 25 |  |  |
|  | No | 2 | 7 | 3 | 12 | 0.35 | 0.90 |
| Years of BD evolution | < 5 | 2 | 6 | 2 | 10 |  |  |
|  | 5-13 | 2 | 4 | 4 | 10 |  |  |
|  | 14-22 | 1 | 6 | 3 | 10 |  |  |
|  | >22 | 2 | 3 | 2 | 7 | 2.09 | 0.94 |
